# Direct aortic approach for TAVI: a single centre experience

**DOI:** 10.1186/1749-8090-8-S1-O316

**Published:** 2013-09-11

**Authors:** H Amrane, F Porta, S Head, A van Boven, A Kappetein

**Affiliations:** 1 Department of Cardiothoracic Surgery, Medisch Centrum Leeuwarden, Leeuwarden, the Netherlands; 2 Department of Cardiothoracic Surgery, Erasmus Medisch Centrum, Rotterdam, the Netherlands; 3 Department of Cardiology, Medisch Centrum Leeuwarden, Leeuwarden, the Netherlands

## Background

Direct aortic approach is an attractive alternative technique for transcatheter aortic valve implantation in challenging patients. We report a brief description of this surgical technique and the short term results of our first series.

## Methods

Our group consisted of 35 inoperable patients presenting aortic valve disease unsuitable for transfemoral valve implantation due to extreme peripheral vessel disease. Data were prospectively collected and retrospectively analysed. Using an upper-J mini sternotomy access to the third intercostal space the ascending aorta was exposed. After placing two purse-strings on the ascending aorta at least 7 cm above annulus, the delivery system was inserted and advanced under fluoroscopy to the optimal position and the valve was deployed.

## Results

Mean age of the patients was 77 years old, mean logistic EuroScore 27 %. Evaluation of the results in these 35 patients treated with the Direct Aortic Approach was made according to the Valve Academic Research Consortium consensus criteria. These include device success endpoints and combined safety endpoints at 30 days. Device success was achieved in 94% of the patients. All cause mortality was 9% at 30 days. One major stroke (3%) and one life threatening bleeding occurred (3%). Pacemaker implantation rate was 9%. The evaluation of the combined efficacy endpoints at 1 year has not been completed yet.

## Conclusion

These results represent the initial experience of a single centre after adopting the direct aortic approach for TAVI as alternative to the transfemoral route. It is less invasive, fast and safe and can be performed with acceptable mortality and morbidity in high risk patients.

Due to the short distance between access point and aortic annulus the positioning of the valve is believed to be more accurate than alternative access route like the transfemoral approach.

